# Deciphering core microbiota in rhizosphere soil and roots of healthy and *Rhizoctonia solani*-infected potato plants from various locations

**DOI:** 10.3389/fmicb.2024.1386417

**Published:** 2024-03-22

**Authors:** Yingmei Yang, Jiang Hu, Xiai Wei, Kai Huang, Chengyun Li, Genhua Yang

**Affiliations:** State Key Laboratory for Protection and Utilization of Bio-Resources in Yunnan, Yunnan Agricultural University, Kunming, Yunnan, China

**Keywords:** disease resistance, microbial diversity, plant components, potato black scurf, soil-borne diseases

## Abstract

Black scurf caused by *Rhizoctonia solani* severely affects potato production. Through amplification of V3-V4 and ITS1-5f variable regions of 16S and internal transcribed spacer (ITS) rRNA, the study was based on the location (Kunming, Qujing, and Zhaotong), plant components (rhizosphere soil and roots), and sample types (healthy and diseased) to assess the diversity of bacterial and fungal communities. We found plant components significantly influence microbial diversity, with rhizosphere soil being more diverse than roots, and the microbial community in the root is mainly derived from the rhizosphere soil. Moreover, the rhizosphere soil and roots of healthy potato plants exhibit greater microbial diversity compared to those of potato plants infected by *Rhizoctonia solani*. Bacterial phyla Actinobacteriota and Acidobacteriota were enriched in rhizosphere soil compared to that of roots, whereas Proteobacteria and Cyanobacteria showed the opposite trend. Fungal phylum Ascomycota was found in low relative abundance in rhizosphere soil than in roots, whereas Basidiomycota showed the opposite trend. Bacterial genera including *Streptomyces*, *Lysobacter*, *Bacillus*, *Pseudomonas*, *Ensifer*, *Enterobacter*, and the *Rhizobium* group (*Allorhizobium*, *Neorhizobium*, *Pararhizobium*, *Rhizobium*), along with fungal genera such as *Aspergillus*, *Penicillium*, *Purpureocillium*, and *Gibberella moniliformis*, have the potential ability of plant growth promotion and disease resistance. However, most fungal species and some bacterial species are pathogenic to potato and could provide a conducive environment for black scurf infection. Interaction within the bacterial network increased in healthy plants, contrasting with the trend in the fungal network. Our findings indicate that *R. solani* significantly alters potato plant microbial diversity, underscoring the complexity and potential interactions between bacterial and fungal communities for promoting potato plant health and resistance against black scurf.

## Introduction

The soilborne pathogenic fungus *Rhizoctonia solani* Kühn, also known as *Thanatephorus cucumeris*, is a basidiomycete with a large host range and different anastomosis groups (AGs). The pathogen can cause diseases in plant families including Solanaceae, Poaceae, Amaranthaceae, Fabaceae, Brassicaceae, Rubiaceae, Araceae, Malvaceae, Moraceae, and Linaceae (Mayo et al., 2015; Verwaaijen et al., 2017; Ajayi-Oyetunde and Bradley, 2018). It is divided into 14 AGs based on its special genetic and biological characteristics, including AG-1 to AG-13 and a bridging isolate AG-BI (Parissa and Tarighi, 2011), among which AG-1 to AG-13 were reported to cause *Rhizoctonia* disease on potato (Balali et al., 1995; Truter and Wehner, 2004; Yanar et al., 2005; Woodhall et al., 2008; Yang et al., 2015; Gush et al., 2019; Murdock et al., 2019; Yang et al., 2019; Lopez Corrales et al., 2023). It is widely known that *R. solani* AG-3 PT is notably aggressive, primarily causing stem canker or black scurf on potatoes (Balali et al., 1995; Woodhall et al., 2008, 2013; Fiers et al., 2011; Yang et al., 2015).

**Figure 1:**
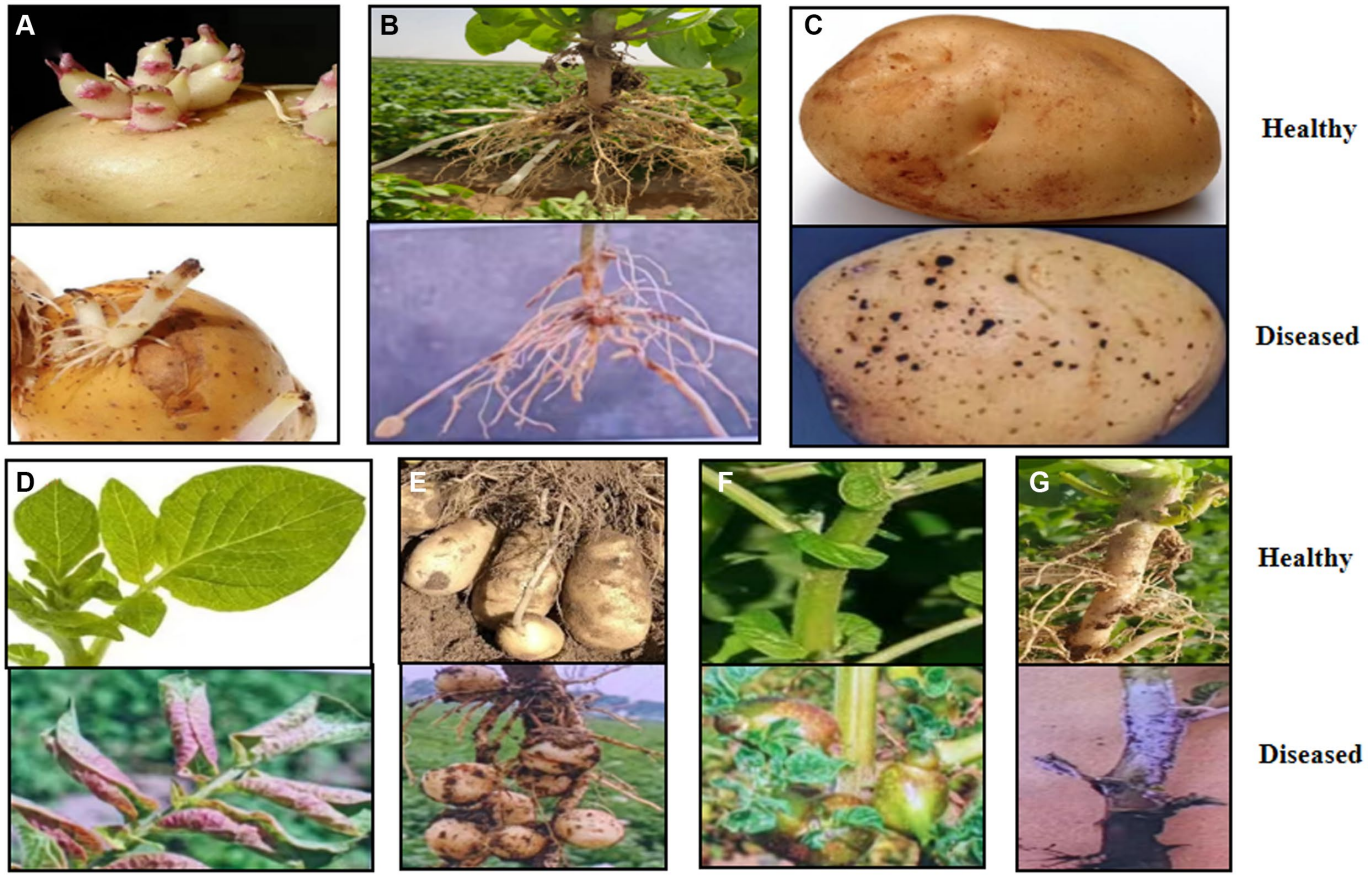
Disease potato plant with typical black scurf symptoms. **(A)** Causing sprout nipping and cankers. **(B)** Sunken brown necrotic lesions formation on stems, stolons, and roots. **(C)** Sclerotia formation on tubers. **(D)** Reddening and inward curling of leaves. **(E)** No tuber formation or small tubers. **(F)** Aerial potato formation on stems. **(G)** White fruiting bodies forming at stem base.

*Rhizoctonia solani* AG-3 PT infects all parts of the potato plant, including sprout, tubers, stems, stolons, roots, and leaves (Atkinson et al., 2010). Infected plants exhibit a range of symptoms such as sprout canker formation (Figure 1A; Gutierrez et al., 1997); sunken, brown, necrotic lesions on stems, stolons, and roots and sclerotia development on tubers (Figures 1B,C; Lehtonen et al., 2009); leaves turning red and upward curling (Figure 1D; Tsror, 2010; Malik et al., 2014); an absence of tuber formation or the growth of small tubers (Figure 1E); aerial tubers formation on stems (Figure 1F; Beukema and van der Zang, 1990); and a grayish-white, felt-like mycelium mat emerging at the base of stems and on the plant parts that are in contact with soil (Figure 1G; Banville and Carling, 2001). All of these symptoms may appear on infected potato plants either separately or in combination (Zheng et al., 2014; Muzhinji et al., 2018). Currently, Black scurf of potato disease occurs in all potato-growing regions around the world, including Yunnan Province, China, leading to marketable yield losses of up to 30% (Banville, 1989; Samsatly et al., 2020; Zrenner et al., 2021). Yunnan Province is located in southwest China, with unique geographical and climatic characteristics (Li et al., 2022). Through an investigation of the occurrence of *Rhizoctonia* diseases in the main potato production areas of Yunnan Province, it was found that the principal production regions, such as the Diqing Tibetan Autonomous Prefecture, Lijiang, and Dali Bai Autonomous Prefecture in Northwestern Yunnan, as well as Zhaotong, Qujing, and Kunming in Northeastern Yunnan, all experienced outbreaks of potato black scurf disease. The incidence rate in fields with severe infections can reach up to 70% to 80%. Particularly in Kunming, Qujing, and Zhaotong, the highest incidence rates were observed at 36.00%, 29.75%, and 29.01%, respectively, followed by Lijiang, Diqing, and Dali, with rates of 27.28%, 20.91%, and 12.36% (Wang et al., 2014). The widespread occurrence of this disease has significantly impacted the yield and quality of local potatoes. Locations play an important role on the occurrence of soilborne diseases (Cai et al., 2021).

It is important to note that *R. solani* AG3 PT predominantly colonizes the belowground parts of potato, including roots (Schreiter et al., 2018). It is a seed- and soil-borne pathogen, which survives through sclerotia and mycelia in infected seeds or soil in tropical environments. Exhibiting facultative parasitism, it can survive in soil residues as a saprophyte without a specific host (Akber et al., 2022). Infected soil is a primary inoculum source, with the pathogen emerging in the rhizosphere soil from roots (Senapati et al., 2022). The spread of the pathogen occurs through mechanisms such as sclerotia dispersal by rain, contaminated soil particles, and mycelial networks connecting plants, as well as via seeds that are already infected (Schreiter et al., 2018). While the teleomorph stage allows airborne basidiospore transmission but is less common in the fields. The sclerotia, the asexual stage, can remain viable in soil for years (Kouzai et al., 2020).

The rhizosphere, the root-surrounding zone, is among Earth’s most intricate ecosystems (Wierzbicka-Woś et al., 2019). Plant disease resistance and growth largely depend on rhizosphere microbial diversity (Mendes et al., 2013; Dong et al., 2019). Plant genetics and soil type play key roles in forming a beneficial rhizosphere microbiome (Mendes et al., 2013; Dong et al., 2019). Recent scientific progress and advanced sequencing technologies have simplified studying plant-microbe interactions (Govindasamy et al., 2014; Mhlongo et al., 2018). Nonetheless, further research is needed to fully understand these interactions and their mechanisms (Bulgarelli et al., 2013).

Currently, biological control involving specific endophytes and rhizosphere microbiome like *Aspergillus*, *Bacillus*, *Pseudomonas*, *Streptomyces*, and *Lysobacter* is a viable method for managing various soilborne diseases and this approach works through mechanisms such as antagonism, altering rhizospheric microbial diversity, and metabolite production (Ma et al., 2018; Schreiter et al., 2018; Wu et al., 2020; Zhang J. et al., 2020; Asaturova et al., 2021; Wei et al., 2021; Abdelaziz et al., 2023; Rashad et al., 2023). In recent years, there have been successive reports on the biological control of potato black scurf. Ikeda et al. (2012) found that *Pythium oligandrum* effectively reduces potato black scurf through mechanisms like hyperparasitism and triggering the plant’s resistance. Furthermore, Tsror et al. (2001) observed that in organic potato cultivation, applying Trichoderma harzianum, nonpathogenic Rhizoctonia (np-R), and cattle manure compost amendment (CMC-H) directly into the soil furrows effectively reduced the occurrence of black scurf. Additionally, studies have shown that strains StS3 and StT2 of Pseudomonas spp. are promising as biocontrol agents. They not only enhance plant growth but also effectively diminish the occurrence of black scurf in potatoes (Tariq et al., 2010). The challenge in effectively controlling this disease stems from its ability to infect a wide variety of hosts, its complex species characteristics, extensive geographical spread, and its resilience (Mayo et al., 2015; Verwaaijen et al., 2017; Ajayi-Oyetunde and Bradley, 2018). Currently, there is no comprehensive solution to fully manage this widespread and stubborn disease.

Potato is a major staple food and the fourth largest crop grown worldwide (Liu et al., 2016). It serves not only as a food source, consumable as a vegetable or processed into snack foods, but also plays a vital role as an industrial raw material (Kowalczewski et al., 2019). The primary regions for potato cultivation in China include areas like Inner Mongolia, Gansu, Guizhou, and Yunnan, which provide ideal natural conditions for its growth. However, *R. solani* annually causes substantial decreases in both the yield and quality of potato crops. Addressing this challenge necessitates a comprehensive understanding of both the microbiome’s population dynamics and its distribution within potato plants, to effectively control the black scurf disease. Therefore, the present study aims to explore the core microbiota (bacteria and fungi) associated with different locations (Kunming, Qujing, and Zhaotong), plant components (rhizosphere soil and roots), and sample types (healthy and diseased). This study posits that exploring the natural potato microbiome can aid in developing strategies to reduce the prevalence of black scurf disease in potatoes.

## Materials and methods

### Sample collection

**Figure 2:**
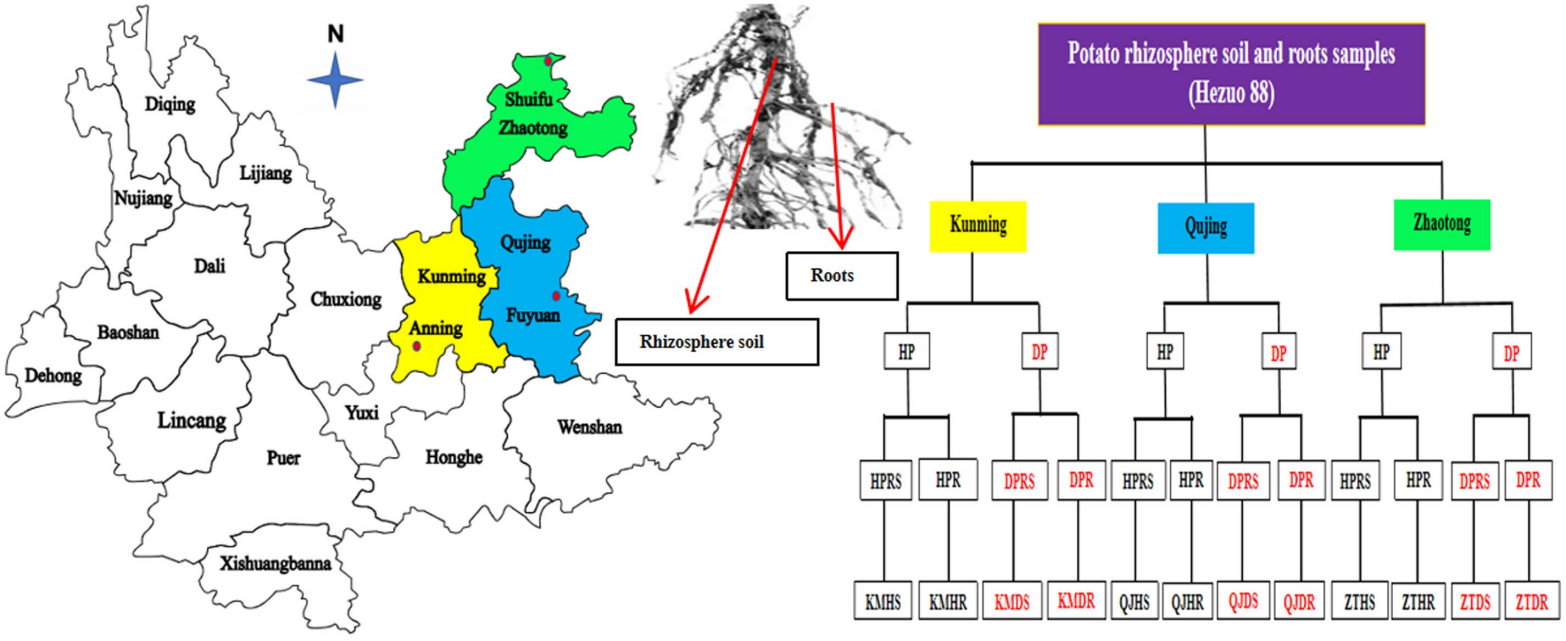
Overview of sampling strategy for both healthy and black scurf-infected diseased Hezuo 88 potato plants. We gathered samples of rhizosphere soil and roots from three distinct sites in Yunnan Province: Kunming (Anning), Qujing (Fuyuan), and Zhaotong (Shuifu). Here: Kunming (KM), Qujing (QJ), Zhaotong (ZT), Healthy plants (HP), Diseased plants (DP), Healthy plants rhizosphere soil (HPRS), Healthy plants roots (HPR), Diseased plants rhizosphere soil (DPRS), Diseased plants roots (DHR), Kunming healthy plants rhizosphere soil (KMHS), Kunming healthy plants roots (KMHR), Kunming diseased plants rhizosphere soil (KMDS), Kunming diseased plants roots (KMDR), Qujing healthy plants rhizosphere soil (QJHS), Qujing healthy plants roots (QJHR), Qujing diseased plants rhizosphere soil (QJDS), Qujing diseased plants roots (QJDR), Zhaotong healthy plants rhizosphere soil (ZTHS), Zhaotong healthy plants roots (ZTHR), Zhaotong diseased plants rhizosphere soil (ZTDS), Zhaotong diseased plants roots (ZTDR).

Rhizosphere soil and root samples of healthy and diseased potato plants were collected from three various locations: Kunming (24.9195°N, 102.4785°E), Qujing (25.6742°N, 104.2550°E), and Zhaotong (28.6299°N, 104.4160°E) in Yunnan Province, China, in September 2021 (Figure 2). Over the past 4 years, these agricultural fields have been consistently used for potato cultivation, employing conventional management practices without the use of any probiotic microorganisms. During the collection process, the top layer of soil, measuring 4–5 cm, was removed, and the potato plants were then carefully uprooted (three plants per field from three different fields for both healthy and diseased plants). The bulk soil was separated by gently shaking the roots, and the finer soil particles adhering to the roots were retained as samples of rhizosphere soil, along with fibrous root samples. In total, 12 composite samples were acquired (three replicates per sample) from these locations in Yunnan. These samples were immediately placed in polythene bags and stored in an icebox for transport. Upon arrival at the laboratory, they were preserved at −80°C for subsequent analysis.

### DNA extraction and polymerase chain reaction amplification

Genomic DNA was isolated from each sample using the Soil and Plant DNA Extraction Kit (Zymo Research Corp., Irvine, CA, United States). The process involved extracting DNA from 0.5 grams of soil and 1 gram of roots per sample, adhering to the kit’s provided guidelines. The DNA’s purity was then assessed using a NanoDrop spectrophotometer (ND2000, Thermo Scientific, Madison, WI, United States), ensuring an optical density (OD) ratio of 260/280 nm between 1.7 and 1.9. The isolated DNA was subsequently stored at −20°C for later analysis. For investigating the bacterial and fungal diversity, the V3-V4 and ITS1-5F regions of the 16S and internal transcribed spacer (ITS) rRNA genes were amplified. This was done using two sets of universal primers: 341F (5′-CCTAYGGGRBGCASCAG-3′) and 806R (5′-GGACTACNNGGGTATCTAAT-3′) for bacteria, and 1743F (5′-GGAAGTAAAAGTCGTAACAAGG-3′) and 2043R (5′-GCTGCGTTCTTCATCGATGC-3′) for fungi (Zhang J. et al., 2020).

### Library preparation and sequencing

The construction of the amplicon library followed the protocols for 16S and ITS Metagenomic Sequencing Library preparation, utilizing the Nextera XT Index Kit (Illumina Inc. Madison, WI, United States). To evaluate the quality of the amplicons, gel electrophoresis was employed. The purification of the amplicon library was conducted using 1X AMPure XP beads, with further assessment on an Agilent DNA1000 chip via a Bioanalyzer2100. Quantification was performed using the Qubit Fluorometer 2.0 and a Qubit dsDNA assay kit (Life Technologies, Cat. No. Q328520; Gao et al., 2018). Equal amounts of these purified amplicons were combined for further sequencing analysis. The sequencing was carried out using the Illumina MiSeq platform at Novogene Bioinformatics Technology Co. Ltd., based in Beijing, China.

### Quality control

The initial sequencing data were gathered in FASTQ format, and the Trimmomatic software was employed to eliminate low-quality reads (those with a score below 20) and to arrange the reads into paired ends (Bolger et al., 2014). For the assembly of these paired-end reads, FLASH software was utilized, setting parameters for a minimum and maximum overlap of 10 and 200 bp, respectively, with a maximum mismatch rate of 20%. The UCHIME software was then applied for the exclusion of chimeric sequences, facilitating the generation of clean reads (Edgar et al., 2011).

### Data processing

The UPARSE pipeline was utilized to process the clean reads, leading to the formation of operational taxonomic units (OTUs) at a similarity threshold of 97% or higher (Edgar, 2013). Taxonomic classification of each representative read and OTU was carried out using the ribosomal database project (RDP) classifier within the SILVA database for bacterial species (with a confidence level of 70%) and the UNITE database for fungal species (Ikeda et al., 2012; Kõljalg et al., 2013). The analysis of OTUs included their relative abundance at both the genus and phylum levels. Furthermore, alpha and beta diversity indices were computed to assess species richness and evenness. To identify common and unique OTUs across different variables, such as sample types, plant components, and locations, a Venn diagram was employed.

### Statistical analysis

Statistical analysis of the data was carried out using the t-test method, setting the significance level at *p* < 0.05. IBM SPSS software, version 20.0 (SPSS Inc., Chicago, IL, United States), was the tool of choice for all statistical computations. The QIIME software, version 1.9.1, was employed to determine various indices, including observed OTUs, Chao1, Shannon, and the abundance-based coverage estimator (ACE). For the beta diversity assessment of both bacterial and fungal communities, the Bray–Curtis dissimilarity index was calculated and then applied in principal coordinate analysis (PCoA) using QIIME. The creation of relative abundance bar plots and heatmaps at the genera level, as well as bar plots at the species level, was achieved through R scripts in R software (version 2.15.3; Dong et al., 2018). Co-occurrence network analysis at the genera level for OTUs was conducted using the SPARCC method in R, adhering to criteria of *p* < 0.05 and a correlation coefficient greater than 0.3. Network characteristics were computed and visualized utilizing Gephi version 0.9.2. For the processing and illustration of all figures, Adobe Illustrator CC 2019, provided by Adobe Systems Inc. in San Francisco, CA, United States, was used.

## Results

### General characteristics of potato microbiome

This study delved into the composition of bacterial and fungal communities in various segments of the potato plant, including the rhizosphere soil and roots, across different regions (Kunming, Qujing, and Zhaotong), and between contrasting types of samples (healthy vs. diseased). Supplementary Table 1 displays information on the number of raw and clean reads, along with quality control metrics (Q20% and Q30%), derived from amplifying the 16S (V3-V4) and ITS (1-5f) rRNA sequences of bacteria and fungi, respectively. Post quality assurance and the removal of chimeric sequences, the average yield was 70,327 bacterial and 77,241 fungal clean reads for each sample, with an average sequence length of 415 bps for bacteria and 265 bps for fungi, as obtained through Illumina sequencing (Supplementary Table 1). Furthermore, rarefaction curves constructed from the OTUs indicated comprehensive sampling coverage for all samples in both the bacterial and fungal communities (Supplementary Figure 1).

### Effects of different locations, plant components, and sample types on beta diversity

**Figure 3:**
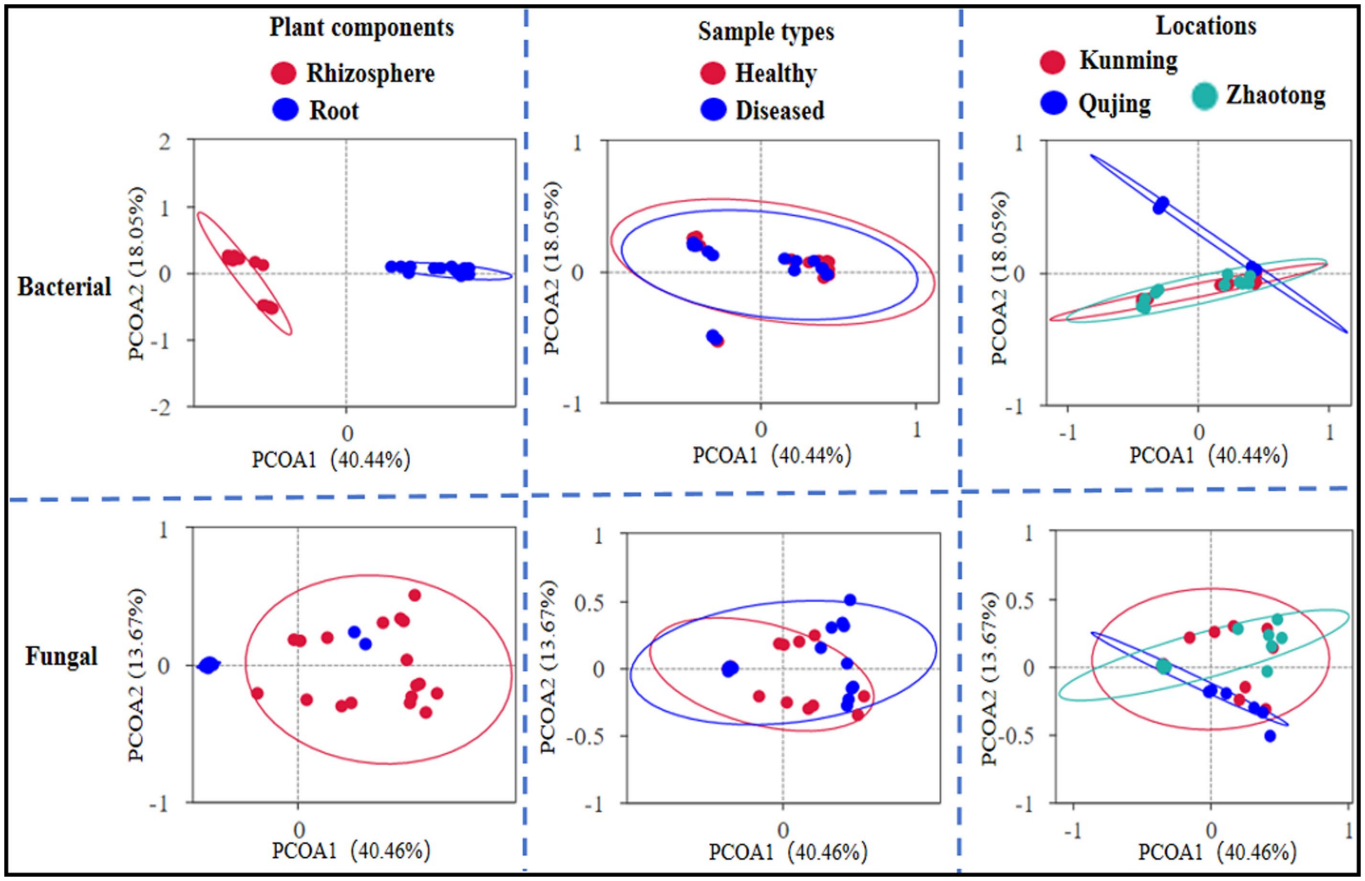
Principal coordinate analysis (PCoA) utilized Bray–Curtis dissimilarity metrics to exhibit the beta diversity analysis across all 12 combined samples (with each sample having three replicates) of potato plants, considering three different variables.

The impact of various factors, including geographical locations (Kunming, Qujing, and Zhaotong), parts of the plant (rhizosphere soil and roots), and the nature of the samples (healthy vs. diseased), on the composition of bacterial and fungal communities was examined. The Bray–Curtis dissimilarity metric was employed to assess beta diversity, which reflects variations in the structure of bacterial and fungal communities, across all 12 composite samples (Figure 3). Among these factors, the components of the plant, namely rhizosphere soil and roots, were found to have a significant effect on the composition of both bacterial and fungal communities. The distinct separation of samples along one axis for the bacterial community composition suggests a more pronounced influence of this variable on the structure of bacterial communities compared to fungal ones. The types of samples and their locations exhibited a minimal impact on both bacterial and fungal communities. A different trend was noted in the principal coordinate analysis (PCoA), revealing differences of 40.44% and 40.46% in the composition of bacterial and fungal communities, respectively. To further evaluate beta diversity, distance heatmap graphs were created for all 12 samples using both Weighted UniFrac (which considers taxa abundances) and Unweighted UniFrac (sensitive to less common taxa), providing insight into the diversity of bacterial and fungal communities (Supplementary Figure 2).

### Effects of different locations, plant components, and sample types on alpha diversity

**Figure 4:**
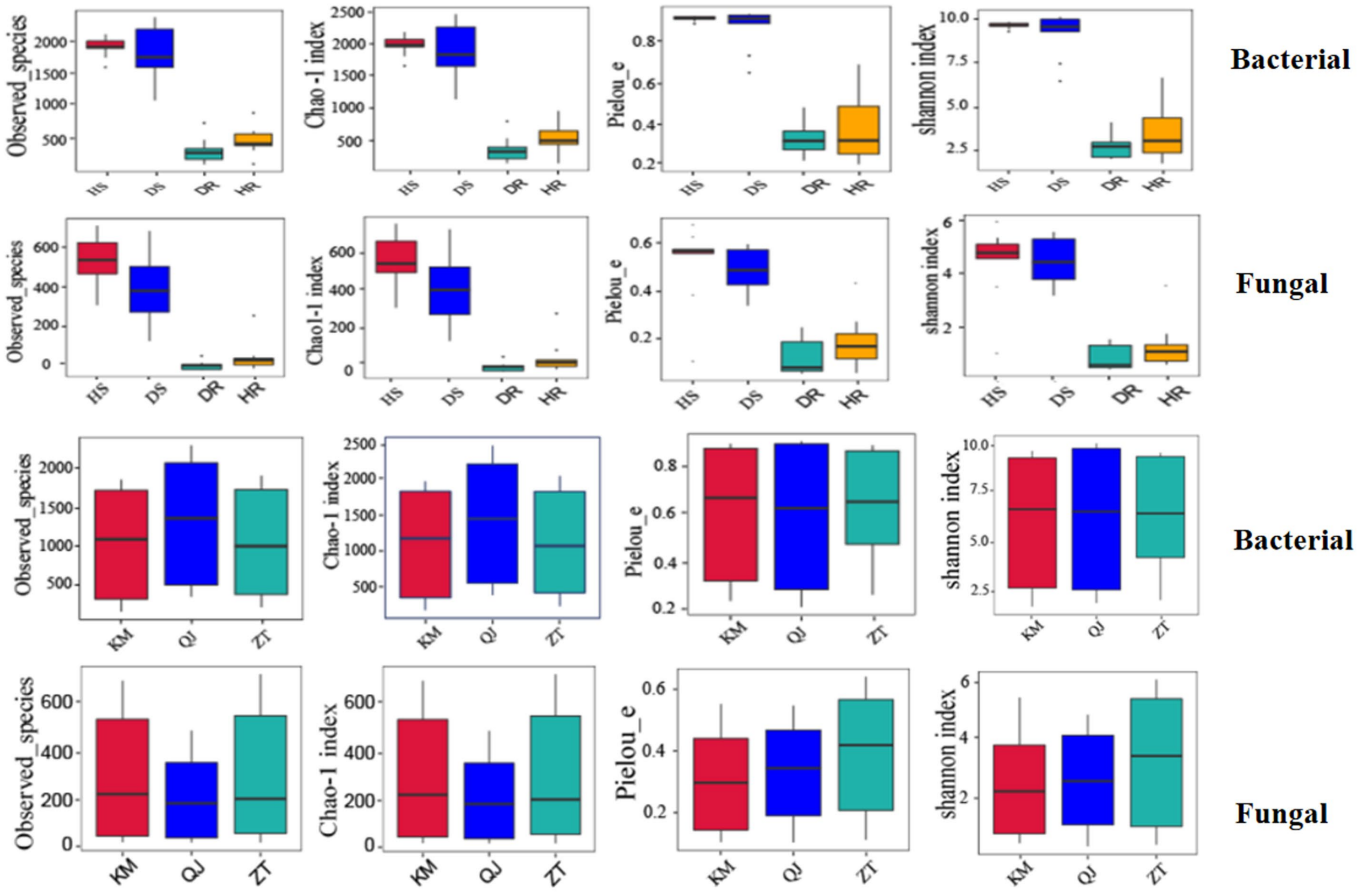
Boxplot of bacterial (top) and fungal (bottom) showing alpha diversity indexes of potato plants under three variables (locations, sample types, and plant components). HS, healthy rhizosphere soil; DS, diseased rhizosphere soil; HR, healthy roots; DR, diseased roots; KM, Kunming; QJ, Qujing; ZT, Zhaotong.

Figure 4 presents the alpha diversity indices, including the Observed species, Shannon, Chao 1, and Pielou_e at cutoff levels of 3%. Within the different plant parts (rhizosphere soil and roots), it was observed that the alpha diversity indices, namely Observed species, Shannon, Chao 1, and Pielou_e, were significantly greater in rhizosphere soil compared to the roots for both bacterial and fungal communities. This suggests a notably higher count of bacteria and fungi in the rhizosphere soil than in the roots. Regarding the types of samples (healthy and diseased), the alpha diversity indices for bacterial and fungal communities were found to be higher in healthy samples as opposed to diseased ones. The locations have little impact on alpha diversity indices of bacterial and fungal communities.

### Analysis of operational taxonomy units

**Figure 5:**
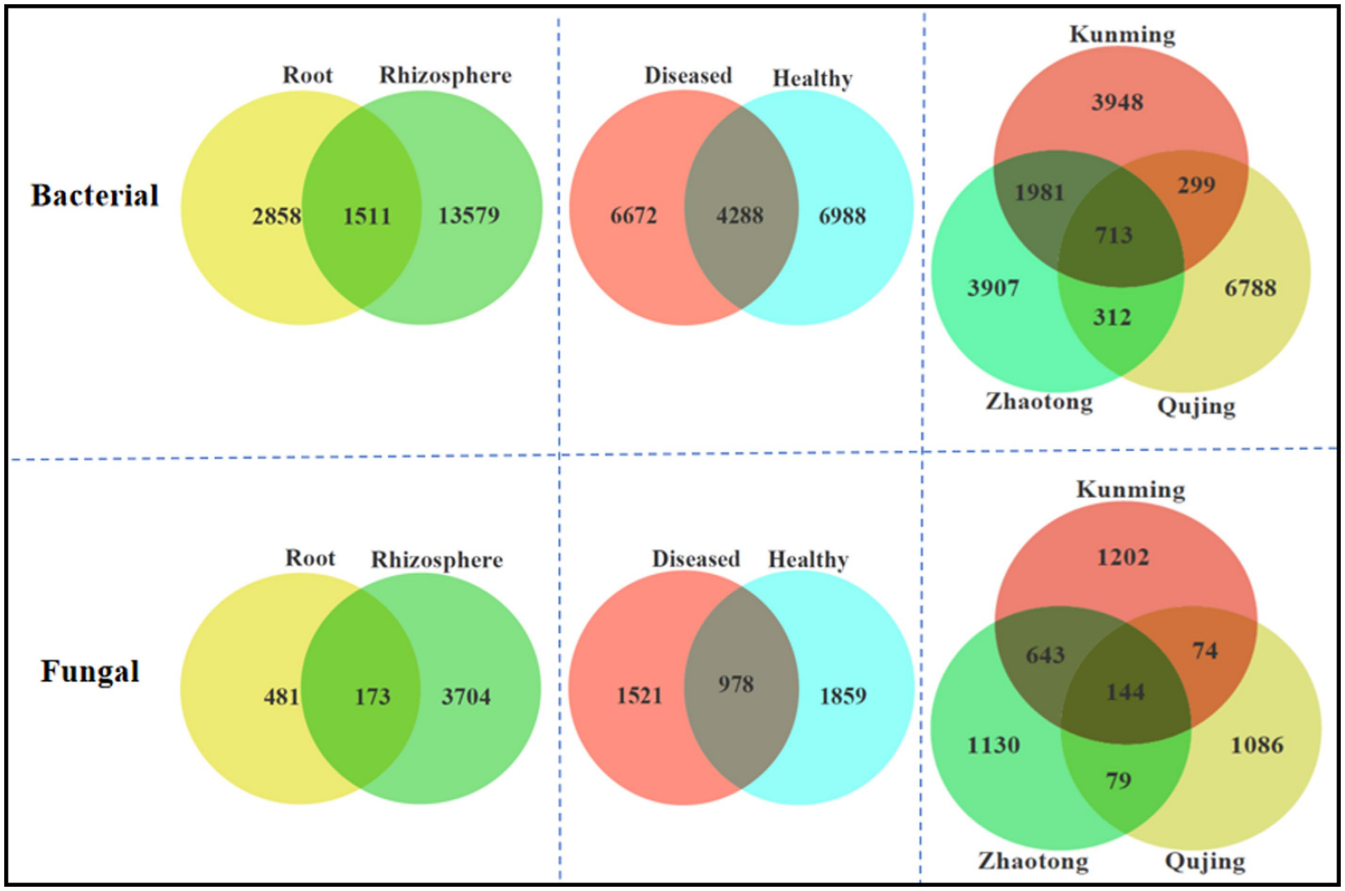
Distribution of bacterial (top) and fungal (bottom) operational taxonomic units in three variables, i.e., plant components (rhizosphere soil and roots), sample types (healthy and diseased), and locations (Kunming, Qujing, and Zhaotong).

The number of operational taxonomic units (OTUs) was observed to be higher in rhizosphere soil than in root samples. Rhizosphere soil samples displayed greater diversity and richness in both bacterial and fungal OTUs compared to root samples. Furthermore, a higher diversity of bacterial communities was noted in healthy plant samples as opposed to diseased ones (Figure 5). In terms of specific bacterial OTUs, rhizosphere soil contained a substantially larger count (13,579) than root tissue (2,858), with 1,511 OTUs shared between them. Conversely, root tissues fewer specific fungal OTUs (481) compared to those in rhizosphere soil (3,704), with 173 OTUs common to both. When comparing healthy and diseased plant samples, unique bacterial OTUs numbered 6,988 and 6,672, respectively, with 4,288 common OTUs between them. For fungal OTUs, healthy samples had 1,859 unique OTUs, surpassing the 1,521 in diseased samples, with 978 OTUs shared. Further analysis of OTUs from different locations (Kunming, Qujing, and Zhaotong) showed that a total of 17,948 and 4,358 specific OTUs were recovered for both bacterial and fungal communities, respectively, and 713 (bacterial) and 144 (fungal) OTUs were found as common OTUs.

### Bacterial and fungal community composition at phylum level

**Figure 6:**
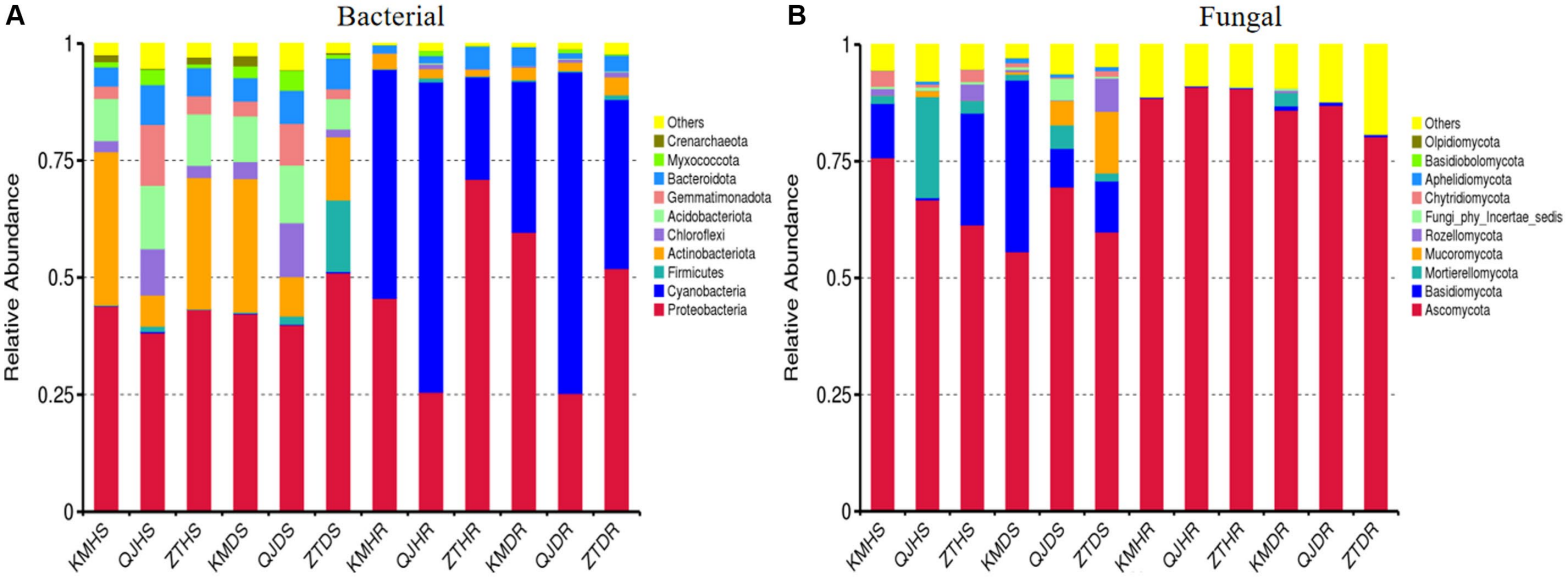
Relative abundance bar plots at phylum level based on the species annotation results in 12 composite samples (average of three replicates per sample) of potato plants under three variables. **(A)** Relative abundance at the phylum level in bacterial communities and **(B)** relative abundance at the phylum level in fungal communities. KM, Kunming; QJ, Qujing; ZT, Zhaotong; HS, healthy rhizosphere soil; DS, diseased rhizosphere soil; HR, healthy roots; DR, diseased roots.

Figure 6 and Supplementary Table 2 display the top 10 bacterial and fungal phyla whose relative abundance exceeds 1%. The predominant bacterial phyla in all samples of rhizosphere soil and roots, with a relative abundance over 1%, include Proteobacteria, Cyanobacteria, Firmicutes, Actinobacteriota, Chloroflexi, Acidobacteriota, Gemmatimonadota, Bacteroidota, Myxococcota, Crenarchaeota, and Others (Figure 6A). Similarly, the leading fungal phyla in these samples, with a relative abundance above 1%, are Ascomycota, Basidiomycota, Mortierellomycota, Mucoromycota, Rozellomycota, Fungi_phy_Incertae_sedis, Chytridiomycota, Aphelidiomycota, Basidiobolomycota, Olpidiomycota, and Others (Figure 6B). Among the three factors (plant components, sample types, and locations), the plant components, namely rhizosphere soil and roots, markedly affect the composition of bacterial and fungal communities. In rhizosphere soil, a high relative abundance of the phyla Actinobacteriota (19.64% average) and Acidobacteriota (10.35% average) was observed. Conversely, in root samples, Proteobacteria (46.54% average) and Cyanobacteria (45.67% average) were more abundantly present (Figure 6A). For fungal phyla, Ascomycota showed a lower relative abundance in rhizosphere soil (64.84% average) compared to roots (87.22% average). On the other hand, the phylum Basidiomycota demonstrated a higher relative abundance in rhizosphere soil (15.33% average) compared to roots (0.48% average; Figure 6B).

### Bacterial and fungal community composition at genera level

**Figure 7:**
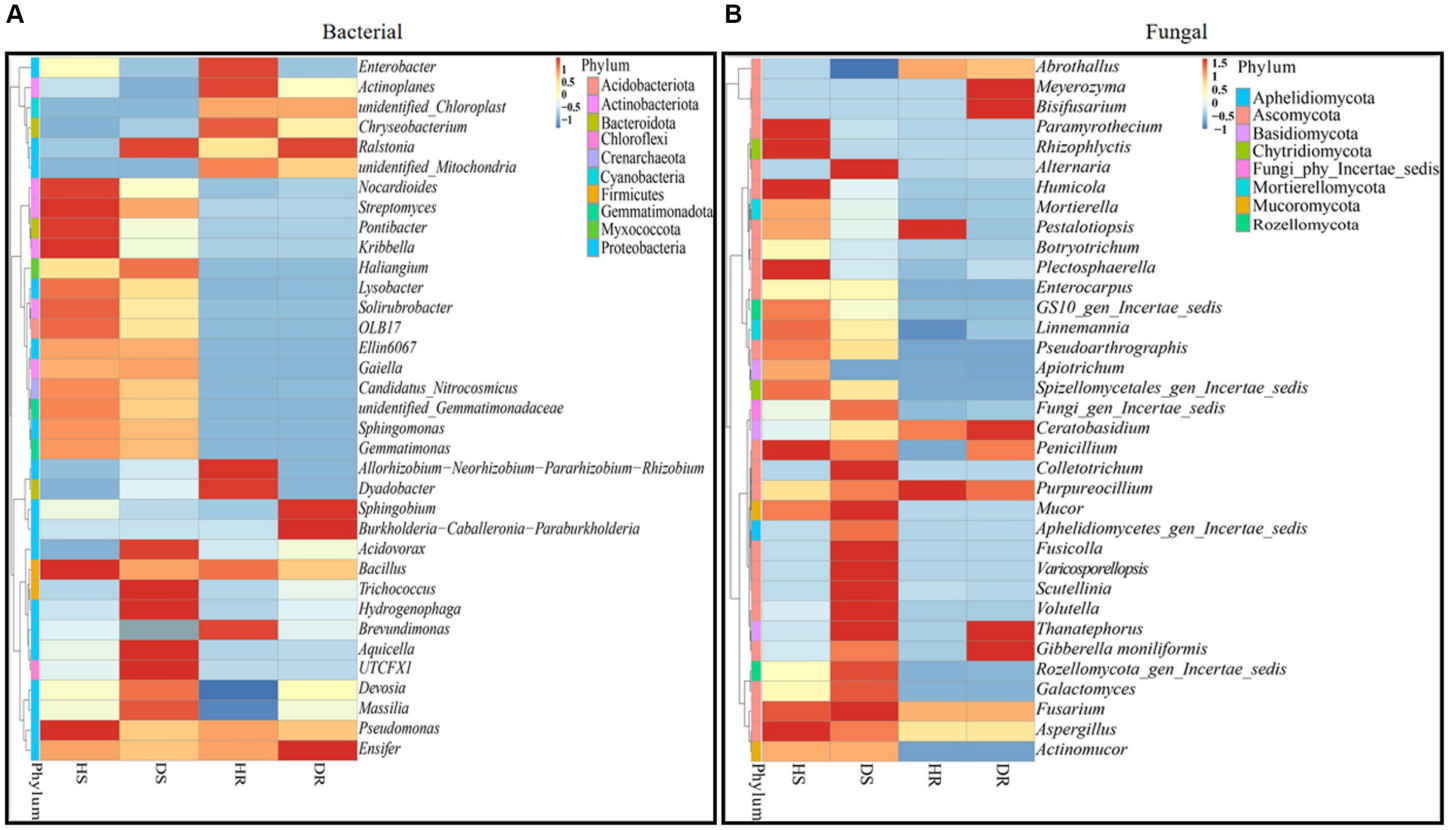
Relative abundance heatmaps at the genus level for top 35 bacterial **(A)** and fungal **(B)** genera in group-wise comparison under three variables. HS, healthy rhizosphere soil; DS, diseased rhizosphere soil; HR, healthy roots; DS, diseased roots.

In our study, we assessed the top 35 bacterial and fungal genera from the previously identified leading 10 phyla. Based on their relative abundance across all samples, these 35 genera were chosen for a heatmap analysis to ascertain their prevalence in varying sample types (healthy and diseased). The heatmaps, illustrating the relative abundance of these top 35 bacterial and fungal genera, are categorized and compared across three different factors: locations, plant components, and sample types, as presented in Figure 7. In healthy rhizosphere soil (HS), genera *Nocardioides*, *Streptomyces*, *Pontibacter*, *Kribbella*, *Lysobacter*, *Bacillus*, and *Pseudomonas* showed high abundance. In diseased rhizosphere soil (DS), genera *Aquicella*, *Hydrogenophaga*, *Trichococcus*, and *Acidovorax* were more prevalent. In healthy roots (HR), genera including *Enterobacter*, *Actinoplanes*, *Chryseobacterium*, *Allorhizobium-Neorhizobium-Pararhizobium-Rhizobium*, *Dyadobacter*, and *Brevundimonas* were dominant, while in diseased roots (DR), *Sphingobium*, *Burkholderia-Caballeronia-Paraburkholderia*, and *Ensifer* were notably abundant. Notably, *Ralstonia* genus exhibited high abundance in both diseased rhizosphere soil and root samples (Figure 7A). Similarly, for fungi, genera like *Paramyrothecium*, *Rhizophlyctis*, *Humicola*, *Plectosphaerella*, *Penicillium*, and *Aspergillus* in HS; *Alternaria*, *Colletotrichum*, *Mucor*, *Fusicolla*, Var*icosporellopsis*, *Scutellinia*, *Volutella*, and *Fusarium* in DS; *Pestalotiopsis*, and *Purpureocillium* in HR; *Meyerozyma*, *Bisifusarium*, *Ceratobasidium*, and *Gibberella moniliformis* in DR, were highly abundant. The genus *Thanatephorus*, however, was particularly abundant in diseased rhizosphere soil and root samples (Figure 7B).

### Relative abundance of bacterial and fungal communities at the species level

**Figure 8:**
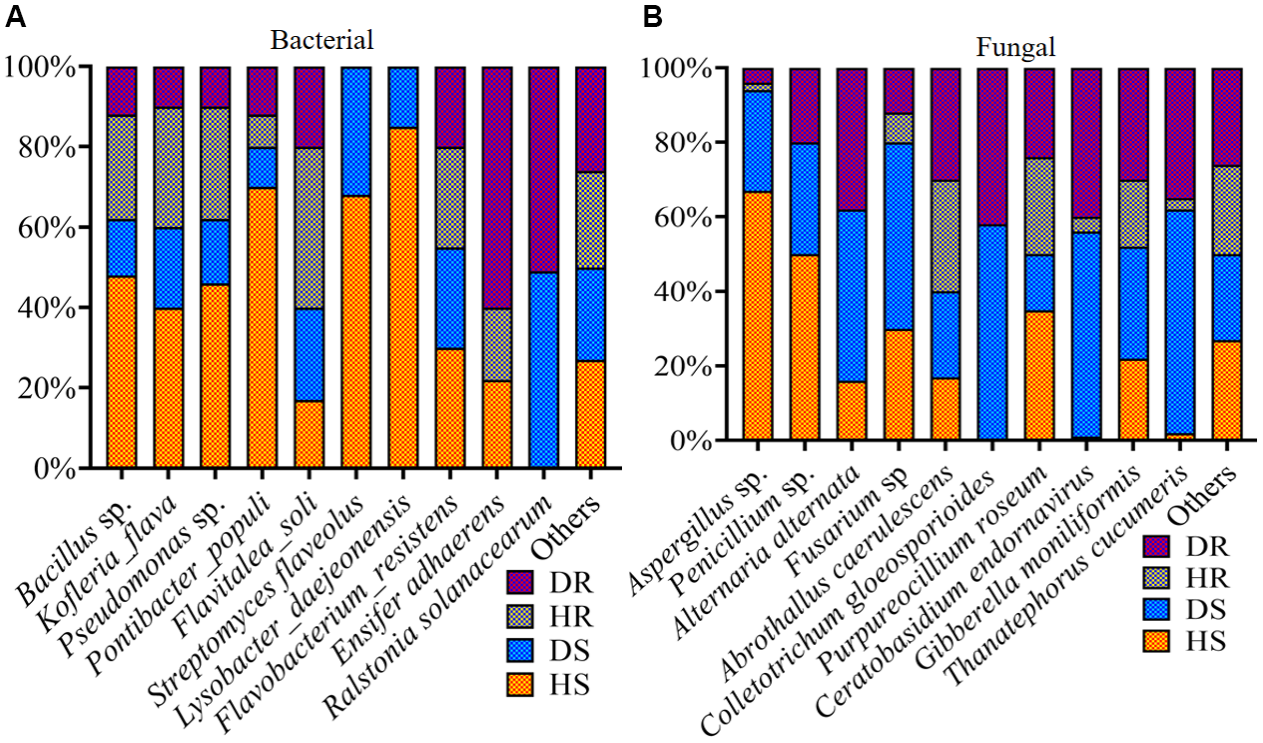
Relative abundance bar plots for top 10 bacterial **(A)** and fungal **(B)** species in group-wise comparison under three variables. HS, healthy rhizosphere soil; DS, diseased rhizosphere soil; HR, healthy roots; DS, diseased roots.

Our analysis focused on the relative abundance of the leading 10 bacterial and fungal species within the top 35 genera. Bar plots depicting the relative abundance of these top 10 bacteria and fungi, categorized according to three criteria (locations, plant components, and sample types), are illustrated in Figure 8. In diseased roots and rhizosphere soil, the bacterial wilt pathogen *Ralstonia solanacearum* was notably prevalent. High abundances of *Bacillus* sp., *Pseudomonas* sp., *Streptomyces flaveolus*, and *Lysobacter*_*daejeonensis* were observed in healthy soil (HS). Furthermore, *Ensifer adhaerens* showed a high prevalence in diseased roots (DR; Figure 8A). In the context of fungi, species like *Aspergillus* sp. and *Penicillium* sp. were predominantly found in HS. Additionally, species such as *Alternaria alternata*, *Thanatephorus cucumeris*, *Colletotrichum gloeosporioides*, *Ceratobasidium endornavirus*, and *Gibberella moniliformis* were particularly abundant in diseased rhizosphere soil and root samples (Figure 8B).

### Characteristics of co-occurrence network

**Figure 9:**
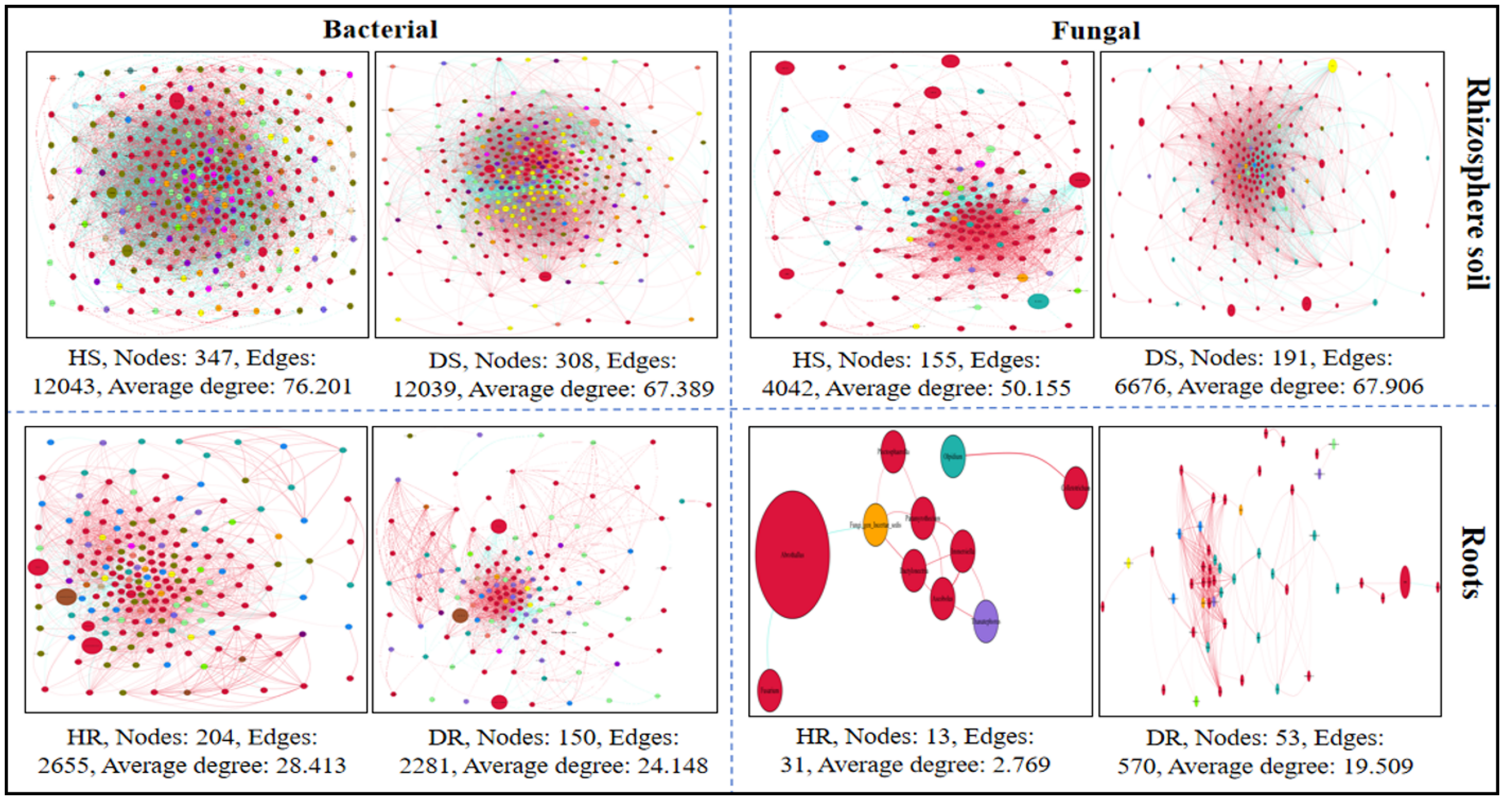
Co-occurrence network analysis of bacterial and fungal communities at genus level associated with healthy and diseased samples. Plant components (rhizosphere soil and roots). Here, HS; healthy soil, DS; diseased soil, HR; healthy roots, DR; diseased roots.

For each healthy and diseased sample, a microbial co-occurrence network at the genus level was developed, encompassing both bacterial and fungal Operational Taxonomic Units (OTUs) and relating to different plant components (rhizosphere soil and roots; Figure 9). Analysis revealed that in the bacterial communities, metrics such as the average degree, the total number of nodes, and the count of edges were more pronounced in healthy samples compared to those in diseased samples. Conversely, in the fungal communities, these same metrics—average degree, number of nodes, and number of edges—were elevated in diseased samples relative to healthy ones. This pattern suggests an enhanced level of interaction and connectivity within the bacterial microbial network in healthy samples, whereas the fungal microbial network exhibited a reverse trend in its connectivity.

## Discussion

The potato’s ability to adapt to various climates has made it crucial for both economy and global food security (Xia et al., 2017; Ajayi-Oyetunde and Bradley, 2018; Iradukunda et al., 2022; Shuang et al., 2022). However, its continuous cultivation has led to an increase in black scurf disease, caused by *Rhizoctonia solani*, affecting potato yields worldwide (Lankau et al., 2020; Zhang X.-Y. et al., 2020; Hiltunen et al., 2021; Kankam et al., 2021; Kiptoo et al., 2021; Qin et al., 2022; Yang et al., 2022). To combat this, strategies such as resistance breeding, crop rotation, adjusting sowing dates and methods, chemical control, and, more recently, the use of disease-suppressive biocontrol agents have been employed (Kiptoo et al., 2021; Zrenner et al., 2021; Munir et al., 2022; Ahmed et al., 2022b). Additionally, the health of soil and microbial diversity in the rhizosphere play vital roles in controlling soilborne diseases and enhancing plant health (Köberl et al., 2013; Dong et al., 2018; Hao and Ashley, 2021).

Repeated cultivation of the same or closely related crops in the same soil, known as monoculture, leads to soil sickness, reducing crop yield and quality (Zheng et al., 2014). The microbiome in the rhizosphere serves as the initial defense mechanism against infections from soilborne pathogens and various forms of abiotic stress (Bulgarelli et al., 2013; Mendes et al., 2013). However, there is limited understanding of the microbial communities in the rhizosphere soil and roots of healthy and black scurf-infected potato plants across various geographical locations. In our study, we conducted a thorough examination of bacterial and fungal communities in different locations, sample types, and plant components by amplifying the V3-V4 and ITS1-5f variable regions of the 16S and internal transcribed spacer (ITS) rRNA genes. Our findings reinforce the notion that distinct plant components markedly influence the bacterial and fungal community structures, regardless of the sample types and geographical locations.

Wang et al. (2022) studied the impact of *Alternaria solani* infection on the microbial communities and multifunctionality in healthy vs. infected potato rhizosphere soils to explore the relationship between soil microbes, functionality, and pathogens. Despite varied observations on how rhizospheric and endophytic bacteria influence plant growth and health, no specific assembly pattern was noted (Lundberg et al., 2012; Lebeis et al., 2015). The rhizosphere is highlighted as a crucial habitat for microbial colonization into plant components, especially bacterial communities (Bulgarelli et al., 2013; Kaushal et al., 2020). In this research, we gathered samples from areas where potatoes have been cultivated consecutively for 4 years, identifying soil, diseased potatoes, and mechanical equipment as key vectors for spreading the black scurf pathogen.

Our study showed that the rhizosphere soil significantly affects microbial diversity, hosting more bacterial and fungal populations than the roots, with most root microbes also present in the rhizosphere across different locations. This suggests root microbes likely originate from the rhizosphere soil, supported by findings that host plants select specific microbial communities for their roots from the rhizosphere (Lundberg et al., 2012). Moreover, we observed also healthy potato plants exhibited richer microbial communities than those infected with black scurf. This may be due to disease stress impacting carbon availability and microbial growth (Kaushal et al., 2020), aligning with research showing healthier soils have more diverse microbes (Fu et al., 2017; Wu et al., 2017). However, some studies report higher microbial diversity in diseased soils (Wu et al., 2021; Zhang et al., 2022), potentially due to factors like soil type, nutrient levels, crop rotation practices, local climate, host plant species, and disease progression (Yuan et al., 2020). Potato plant resistance and severity of *R. solani* in different plant components also contribute to the prevalence of microbial communities. Bacterial and fungal OTU count was recorded maximum in all locations and plant components in rhizosphere soil samples than in the root samples.

Host plant’s environment can either support or hinder the colonization of specific bacterial and fungal groups in various plant components. Thus, bacterial and fungal communities in these specific plant components are either boosted or exhausted (Kaushal et al., 2020). In our study, we found stable core bacterial and fungal communities across all samples, with notable differences in their distribution among plant components. Specifically, Actinobacteriota and Acidobacteriota were more common in the rhizosphere soil, while Proteobacteria and Cyanobacteria were more prevalent in the roots. For fungi, Ascomycota was less abundant in the rhizosphere compared to the roots, whereas Basidiomycota showed the opposite pattern. Results showed that the black scurf pathogen significantly impacts these microbial communities.

A substantial number of varied bacteria and fungi are known to boost plant growth, reduce the occurrence of diseases, and provide other advantageous biological functions for plants (Santoyo et al., 2016; Gouda et al., 2018). Our research identifies core microbial communities that promote plant growth, enhance resistance, and lower disease rates by producing antibiotics, volatile compounds, secondary metabolites, and fixing nitrogen. Notably abundant bacterial genera include *Streptomyces*, *Lysobacter*, *Bacillus*, *Pseudomonas*, *Ensifer*, *Enterobacter*, and the *Rhizobium* group (*Allorhizobium*, *Neorhizobium*, *Pararhizobium*, *Rhizobium*), which are crucial for plant growth, nutrient acquisition, and suppressing diseases. Among fungi, *Gibberella*, *Aspergillus*, *Penicillium* sp., and *Purpureocillium* act as growth enhancers and biocontrol agents, while others may be saprophytic or pathogenic to crops.

The black scurf pathogen, *R. solani* (also known as *Thanatephorus cucumeris*), was found in high concentrations in the rhizosphere soil and roots of diseased plants, posing a significant threat to agriculture by infecting plant roots and disrupting symbiotic relationships with beneficial soil microbes. *R. solani* competes for nutrients and may even release toxins to suppress beneficial microbes like *Streptomyces*, *Lysobacter*, *Bacillus*, *Pseudomonas*, *Ensifer*, *Gibberella*, *Aspergillus*, *Penicillium* sp., and *Purpureocillium*, reducing their population and negatively affecting the soil’s microbial community and plant health. Additionally, pathogenic bacteria and fungi such as *Ralstonia solanacearum*, *Alternaria alternata*, *Thanatephorus cucumeris*, *Colletotrichum gloeosporioides*, and *Ceratobasidium* endornavirus were also present in high relative abundance in diseased rhizosphere soil and roots, indicating that the health of potato plants is closely linked to these microbial communities, with pathogenic ones contributing to the spread of *R. solani* and black scurf disease.

Microbial co-occurrence networks show direct and indirect relationships among microbes, highlighting their roles and ecological niches. Our findings indicate that *R. solani* has a more pronounced effect on the structure of bacterial communities than on fungal communities. This aligns with other research, such as that by Ahmed et al. (2022a), showing *Ralstonia solanacearum’s* significant impact on bacterial structures over fungal ones (Ahmed et al., 2022a). Healthy potato plants showed fewer negative bacterial interactions than diseased ones, echoing the findings of Wang et al. (2021) and Wu et al. (2021). Stronger and more resilient inter-microbial relationships, less vulnerable to environmental shifts, and more effective in suppressing soil-borne diseases (Tao et al., 2018). The heightened competition among microbial taxa in diseased conditions may result from an increase in potential pathogenic fungal and bacterial taxa in the rhizospheres of diseased potatoes. Simultaneously, the plant roots may recruit beneficial microbes to counteract pathogenic fungi (Zhang et al., 2022). Key species identified, such as *Streptomyces*, *Lysobacter*, *Bacillus*, *Pseudomonas*, *Ensifer*, *Enterobacter*, the *Rhizobium* group (*Allorhizobium*, *Neorhizobium*, *Pararhizobium*, *Rhizobium*), *Aspergillus*, *Penicillium*, *Purpureocillium*, and *Gibberella moniliformis*, are recognized for enhancing plant growth and disease resistance (Ma et al., 2018; Schreiter et al., 2018; Wu et al., 2020; Zhang J. et al., 2020; Asaturova et al., 2021; Wei et al., 2021; Abdelaziz et al., 2023; Rashad et al., 2023).

## Conclusion

Our study concludes that potato plants host diverse bacterial and fungal communities, with their composition significantly varying across different plant components. The rhizosphere soil exhibited richer microbial diversity than the roots, and healthy plants showed greater microbial diversity than diseased ones. We found that certain microbes in the potato microbiome contribute to growth and disease resistance, identifying pathogenic communities in diseased plants linked to potato black scurf disease. Future research should focus on the microbiomes of black scurf-resistant potato varieties, exploring potential for disease control through microbiome manipulation to improve yield and quality.

## Data availability statement

The original contributions presented in the study are included in the article/Supplementary material, further inquiries can be directed to the corresponding author.

## Author contributions

YY: Conceptualization, Data curation, Investigation, Methodology, Software, Supervision, Writing – original draft, Writing – review & editing. JH: Data curation, Investigation, Writing – review & editing. XW: Investigation, Writing – review & editing. KH: Investigation, Writing – review & editing. CL: Conceptualization, Formal analysis, Methodology, Writing – review & editing. GY: Conceptualization, Formal Analysis, Funding acquisition, Methodology, Project administration, Resources, Supervision, Validation, Visualization, Writing – review & editing.
