# Supplementary material for: Deciphering core microbiota in rhizosphere soil and roots of healthy and Rhizoctonia solani-infected potato plants from various locations
Source: Front Microbiol. 2024 Mar 22;15:1386417. doi: 10.3389/fmicb.2024.1386417 (PMC10995396; doi:10.3389/fmicb.2024.1386417)
Supplement: Supplementary file 1 [file Table_1.DOCX]

| **Table S1** Data obtained from sequencing data of V3-V4 and ITS1-5f variable regions of 16S and ITS rRNA of bacteria and fungi, respectively | | | | | | |
| --- | --- | --- | --- | --- | --- | --- |
| **Sample Id** | **Raw reads**  **(#)** | **Clean reads**  **(#)** | | **Average (bP)** | **Q20**  **(%)** | **Q30**  **(%)** |
| **Bacteria (16S; V3-V4)** | | | | | | |
| KMHS | 82683±5896.88 | | 56828±5279.14 | 414±0.35 | 98.53 | 95.03 |
| KMDS | 78496±1221.47 | | 53440±1695.85 | 415±1.35 | 98.49 | 94.95 |
| KMHR | 89360±2068.77 | | 85206±2173.38 | 412±4.17 | 98.52 | 94.91 |
| KMDR | 87776±1118.32 | | 82087±1320.08 | 418±8.66 | 98.54 | 94.97 |
| QJHS | 83394±3034.12 | | 57511±3461.80 | 417±0.11 | 98.55 | 95.12 |
| QJDS | 87785±843.12 | | 61060±1326.42 | 417±0.49 | 98.53 | 95.06 |
| QJHR | 90055±4791.24 | | 84522±5262.63 | 408±0.97 | 98.42 | 94.66 |
| QJDR | 87272±1047.99 | | 82447±1010.24 | 407±0.52 | 98.43 | 94.68 |
| ZTHS | 86069±3369.16 | | 60739±2423.73 | 416±0.40 | 98.59 | 95.22 |
| ZTDS | 81226±4054.05 | | 55098±5018.95 | 420±2.68 | 98.50 | 94.91 |
| ZTHR | 89645±3092.62 | | 84158±2709.50 | 420±2.71 | 98.48 | 94.81 |
| ZTDR | 88343±2632.49 | | 80832±1724.36 | 413±2.87 | 98.37 | 94.54 |
| **Fungi (ITS; 1-5f)** | | | | | | |
| KMHS | 90,065±10890.45 | | 75,146± 8191.67 | 261± 10.42 | 97.04 | 91.54 |
| KMDS | 98,885±4429.65 | | 87,804± 2342.49 | 248± 28.58 | 99.32 | 94.47 |
| KMHR | 90,041±3836.71 | | 75,631± 3783.71 | 285± 0.13 | 94.64 | 85.39 |
| KMDR | 88,754±4983.62 | | 76,233± 6738.24 | 275± 15.55 | 95.22 | 86.90 |
| QJHS | 86,939±24611.14 | | 74,661± 21402.28 | 257± 2.82 | 97.79 | 93.37 |
| QJDS | 96,700±343.92 | | 77,352± 2240.57 | 246± 10.66 | 98.90 | 96.35 |
| QJHR | 85,890±673.91 | | 73,783± 365.71 | 284± 1.19 | 94.67 | 85.44 |
| QJDR | 91,019±2976.83 | | 77,443± 2710.93 | 281± 3.60 | 94.73 | 85.65 |
| ZTHS | 99,472±4488.53 | | 84,713± 5857.42 | 238± 13.43 | 98.98 | 96.58 |
| ZTDS | 97,328±2171.44 | | 69,526± 10316.32 | 239± 19.36 | 99.13 | 96.86 |
| ZTHR | 92,148±2214.13 | | 77701± 2497.13 | 285± 0.51 | 94.62 | 85.37 |
| ZTDR | 89,914±3593.84 | | 76,902± 3990.70 | 285± 1.07 | 94.71 | 85.68 |
| Here; Q20 and Q30 are the percentages of bases with base quality values greater than 20 (sequencing error rate less than 1%) and 30 (sequencing error rate less than 0.1%). Data is represented as standard error of means (±SEM, n=3). | | | | | | |

**Table S2** Relative abundance of top 10 dominant bacterial and fungal phyla in different sample

| **Taxonomy** | **KMHS** | **QJHS** | **ZTHS** | **KMDS** | **QJDS** | **ZTDS** |
| --- | --- | --- | --- | --- | --- | --- |
| **Bacteria (16S; V3-V4)** | | | | | | |
| Proteobacteria | 0.4397±  0.0501 | 0.3825±  0.0321 | 0.4318±  0.0135 | 0.4233±  0.0120 | 0.3994±  0.0564 | 0.5105±  0.1790 |
| Cyanobacteria | 0.0008±  0.0008 | 0.0028±  0.0030 | 0.0003±  0.0001 | 0.0013±  0.0014 | 0.0016±  0.0012 | 0.0030±  0.0025 |
| Firmicutes | 0.0015±  0.0025 | 0.0115±  0.0058 | 0.0010±  0.0006 | 0.0025±  0.0026 | 0.0175±  0.0043 | 0.1527±  0.2383 |
| Actinobacteriota | 0.3271±  0.0488 | 0.0665±  0.0088 | 0.2809±  0.0322 | 0.2848±  0.0193 | 0.0840±  0.0194 | 0.1348±  0.0734 |
| Chloroflexi | 0.0237±  0.0043 | 0.0988±  0.0202 | 0.0261±  0.0027 | 0.0363±  0.0088 | 0.1152±  0.0624 | 0.0166±  0.0094 |
| Acidobacteriota | 0.0902±  0.0063 | 0.1355±  0.0222 | 0.1099±  0.0228 | 0.0975±  0.0057 | 0.1231±  0.0137 | 0..0651±  0..0344 |
| Gemmatimonadota | 0.0265±  0.0084 | 0.1301±  0.0211 | 0.0387±  0.0057 | 0.0318±  0.0055 | 0.0889±  0.0124 | 0.0209±  0.0063 |
| Bacteroidota | 0.0408±  0.0140 | 0.0850±  0.0177 | 0.0602±  0.0094 | 0.0500±  0.0151 | 0.0709±  0.0116 | 0.0656±  0.0128 |
| Myxococcota | 0.0111±  0.0032 | 0.0329±  0.0032 | 0.0078±  0.0007 | 0.0252±  0.0110 | 0.0427±  0.0041 | 0.0082±  0.0048 |
| Crenarchaeota | 0.0150±  0.0085 | 0.0020±  0.0007 | 0.0149±  0.0059 | 0.0218±  0.0149 | 0.0012±  0.0009 | 0.0038±  0.0009 |
| **Taxonomy** | **KMHR** | **QJHR** | **ZTHR** | **KMDR** | **QJDR** | **ZTDR** |
| **Bacteria (16S; V3-V4)** | | | | | | |
| Proteobacteria | 0.4562±  0.1317 | 0.2556±  0.0168 | 0.7105±  0.0550 | 0.5971±  0.3361 | 0.2531±  0.0438 | 0.5197±  0.0660 |
| Cyanobacteria | 0.4891±  0.0983 | 0.6625±  0.0565 | 0.2187±  0.0806 | 0.3224±  0.3373 | 0.6865±  0.0677 | 0.3611±  0.0993 |
| Firmicutes | 0.0011±  0.0012 | 0.0087±  0.0124 | 0.0019±  0.0014 | 0.0035±  0.0049 | 0.0029±  0.0011 | 0.0099±  0.0032 |
| Actinobacteriota | 0.0326±  0.0228 | 0.0199±  0.0096 | 0.0140±  0.0111 | 0.0269±  0.0219 | 0.0182±  0.0081 | 0.0384±  0.0354 |
| Chloroflexi | 0.0012±  0.0006 | 0.0098±  0.0079 | 0.0014±  0.0010 | 0.0025±  0.0029 | 0.0066±  0.0032 | 0.0106±  0.0113 |
| Acidobacteriota | 0.0005±  0.0003 | 0.0025±  0.0029 | 0.0002±  0.0003 | 0.0006±  0.0007 | 0.0016±  0.0010 | 0.0018±  0.0015 |
| Gemmatimonadota | 0.00007±  0.00007 | 0.0003±  0.0001 | 0.0002±  0.0002 | 0.0000±  0.0000 | 0.0004±  0.0005 | 0.0001±  0.0002 |
| Bacteroidota | 0.0166±  0.0126 | 0.0154±  0.0089 | 0.0479±  0.0569 | 0.0405±  0.0486 | 0.0116±  0.0049 | 0.0337±  0.0182 |
| Myxococcota | 0.0005±  0.0006 | 0.0110±  0.0006 | 0.0110±  0.0054 | 0.0014±  0.0020 | 0.0003±  0.0004 | 0.0079±  0.0066 |
| Crenarchaeota | 0.0002±  0.00005 | 0.0001±  0.0001 | 0.0001±  0.00004 | 0.0001±  0.0001 | 0.0004±  0.0002 | 0.0005±  0.0005 |
| **Taxonomy** | **KMHS** | **QJHS** | **ZTHS** | **KMDS** | **QJDS** | **ZTDS** |
| **Fungi (ITS; 1-5f)** | | | | | | |
| Ascomycota | 0.7581±  0.0330 | 0.6677±  0.1272 | 0.6141±  0.0908 | 0.5565±  0.3854 | 0.6953±  0.0122 | 0.5989±  0.0863 |
| Basidiomycota | 0.1162±  0.0414 | 0.0048±  0.0039 | 0.2394±  0.1216 | 0.3677±  0.3425 | 0.0826±  0.0407 | 0.1091±  0.0552 |
| Mortierellomycota | 0.0172±  0.0054 | 0.2164±  0.1264 | 0.0271±  0.0041 | 0.0127±  0.0146 | 0.0504±  0.0117 | 0.0172±  0.0051 |
| Mucoromycota | 0.0004±  0.0001 | 0.0125±  0.0127 | 0.0003±  0.0001 | 0.0052±  0.0084 | 0.0520±  0.0356 | 0.1322±  0.0966 |
| Rozellomycota | 0.0142±  0.0110 | 0.0007±  0.0002 | 0.0351±  0.0194 | 0.0049±  0.0036 | 0.0015±  0.0007 | 0.0712±  0.0912 |
| Fungi_phy_  Incertae_sedis | 0.0049±  0.0036 | 0.0073±  0.0016 | 0.0054±  0.0014 | 0.0058±  0.0066 | 0.0461±  0.0246 | 0.0039±  0.0001 |
| Chytridiomycota | 0.0337±  0.0116 | 0.0063±  0.0069 | 0.0257±  0.0034 | 0.0087±  0.0126 | 0.0028±  0.0019 | 0.0120±  0.0101 |
| Aphelidiomycota | 0.0002±  0.0002 | 0.0063±  0.0055 | 0.0003±  0.0003 | 0.0105±  0.0147 | 0.0067±  0.0049 | 0.0087±  0.0061 |
| Basidiobolomycota | 0.0000±  0.0000 | 0.0003±  0.0004 | 0.0000±  0.0000 | 0.0000±  0.0000 | 0.0009±  0.0010 | 0.0000±  0.0000 |
| Olpidiomycota | 0.0005±  0.0005 | 0.0000±  0.0000 | 0.0002±  0.0001 | 0.0004±  0.0003 | 0.0000±  0.0000 | 0.0010±  0.0001 |
| **Taxonomy** | **KMHR** | **QJHR** | **ZTHR** | **KMDR** | **QJDR** | **ZTDR** |
| **Fungi (ITS; 1-5f)** | | | | | | |
| Ascomycota | 0.8852±  0.0546 | 0.9095±  0.0858 | 0.9059±  0.0732 | 0.8597±  0.1470 | 0.8701±  0.0284 | 0.8030±  0.0866 |
| Basidiomycota | 0.0025±  0.0023 | 0.0026±  0.0038 | 0.0024±  0.0028 | 0.0093±  0.0135 | 0.0074±  0.0101 | 0.0043±  0.0033 |
| Mortierellomycota | 0.0001±  0.0001 | 0.0000±  0.0000 | 0.00003±  0.00006 | 0.0286±  0.0494 | 0.0001±  0.0001 | 0.0000±  0.0000 |
| Mucoromycota | 0.0000±  0.0000 | 0.0000±  0.0000 | 0.0000±  0.0000 | 0.0000±  0.0000 | 0.0000±  0.0000 | 0.0000±  0.0000 |
| Rozellomycota | 0.0001±  0.0001 | 0.0000±  0.0000 | 0.0000±  0.0001 | 0.0048±  0.0084 | 0.0000±  0.0000 | 0.0001±  0.0001 |
| Fungi_phy_  Incertae_sedis | 0.0001±  0.0002 | 0.0001±  0.0001 | 0.0020±  0.0034 | 0.0047±  0.0053 | 0.0000±  0.0000 | 0.0003±  0.0004 |
| Chytridiomycota | 0.0000±  0.0000 | 0.0000±  0.0000 | 0.0000±  0.0000 | 0.0002±  0.0004 | 0.0000±  0.0000 | 0.0002±  0.0002 |
| Aphelidiomycota | 0.0000±  0.0000 | 0.0000±  0.0000 | 0.0000±  0.0000 | 0.0000±  0.0000 | 0.0000±  0.0000 | 0.0004±  0.0007 |
| Basidiobolomycota | 0.0000±  0.0000 | 0.0000±  0.0000 | 0.0000±  0.0000 | 0.0000±  0.0000 | 0.0000±  0.0000 | 0.0000±  0.0000 |
| Olpidiomycota | 0.0002±  0.0002 | 0.0000±  0.0000 | 0.0010±  0.0002 | 0.0000±  0.0000 | 0.0000±  0.0000 | 0.0006±  0.0010 |

Relative abundance in bacterial and fungal communities of the top 10 species with highest abundance at phylum level. Data is represented as standard error of means (±SEM, n=3).
