# Supplementary material for: Deciphering core microbiota in rhizosphere soil and roots of healthy and Rhizoctonia solani-infected potato plants from various locations
Source: Front Microbiol. 2024 Mar 22;15:1386417. doi: 10.3389/fmicb.2024.1386417 (PMC10995396; doi:10.3389/fmicb.2024.1386417)
Supplement: Supplementary file 2 [file Presentation_1.PPTX]

## Slide 1
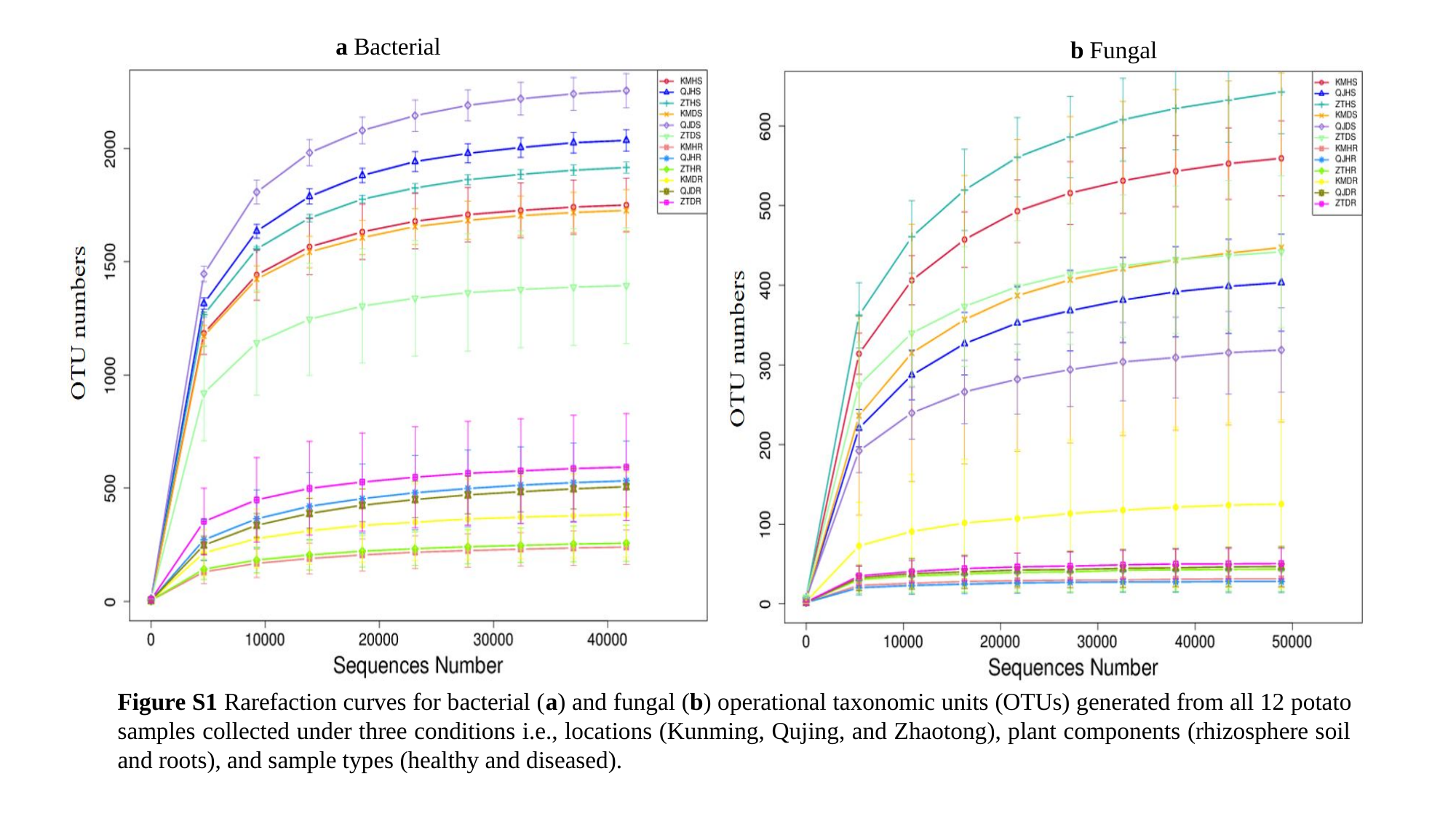

a Bacterial
b Fungal
Figure S1 Rarefaction curves for bacterial (a) and fungal (b) operational taxonomic units (OTUs) generated from all 12 potato samples collected under three conditions i.e., locations (Kunming, Qujing, and Zhaotong), plant components (rhizosphere soil and roots), and sample types (healthy and diseased).

## Slide 2
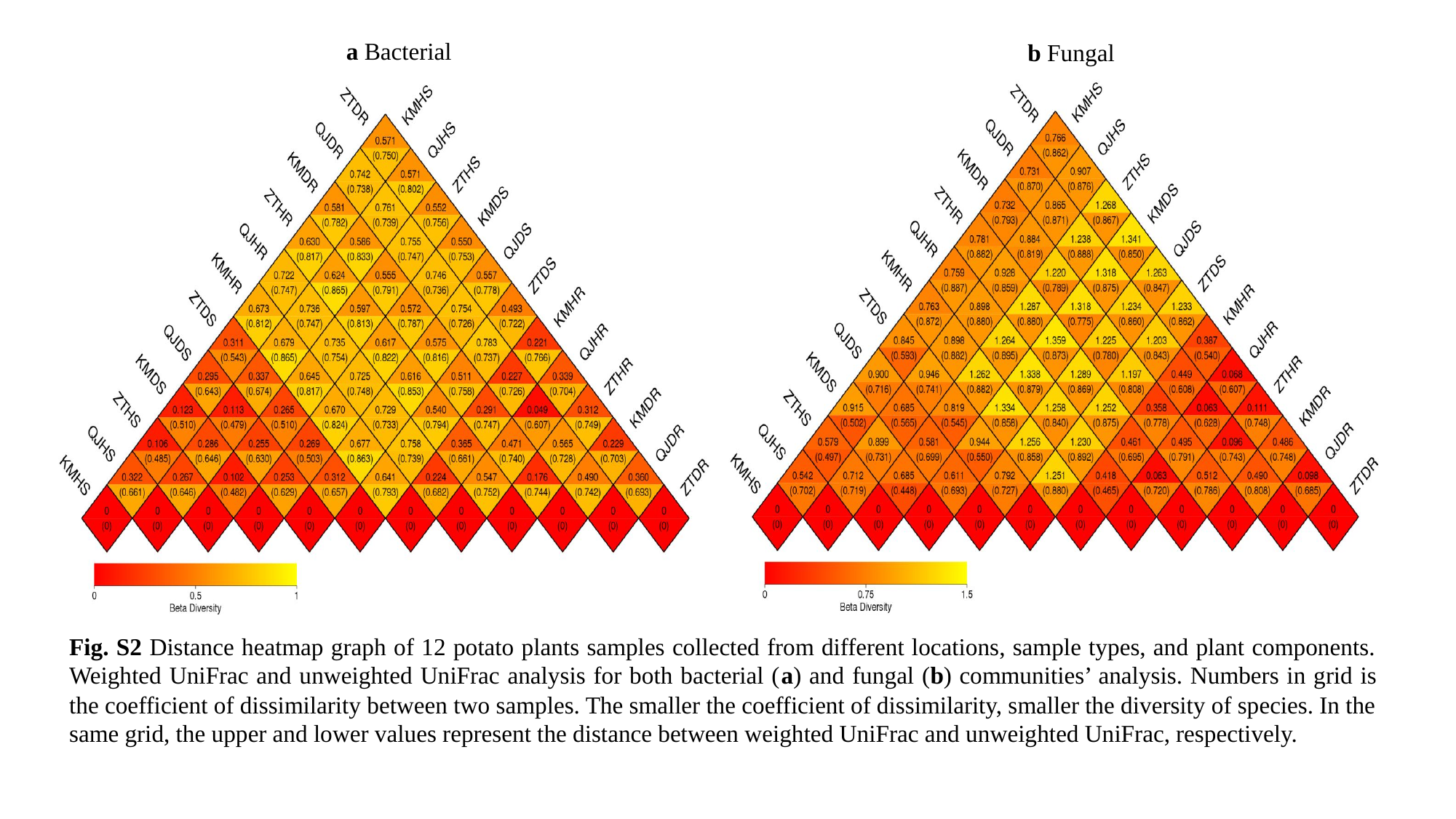

a Bacterial
b Fungal
Fig. S2 Distance heatmap graph of 12 potato plants samples collected from different locations, sample types, and plant components. Weighted UniFrac and unweighted UniFrac analysis for both bacterial (a) and fungal (b) communities’ analysis. Numbers in grid is the coefficient of dissimilarity between two samples. The smaller the coefficient of dissimilarity, smaller the diversity of species. In the same grid, the upper and lower values represent the distance between weighted UniFrac and unweighted UniFrac, respectively.
